# Supplementary material for: Are women with endometriosis more likely to experience reduced physical performance compared to women without the condition?
Source: PeerJ. 2024 Apr 22;12:e16835. doi: 10.7717/peerj.16835 (PMC11044877; doi:10.7717/peerj.16835)
Supplement: Supplemental Information 2 [file peerj-12-16835-s002.docx]

STROBE Statement—checklist of items that should be included in reports of observational studies

|  | Item No. | Recommendation | Page  No. | Relevant text from manuscript |
| --- | --- | --- | --- | --- |
| **Title and abstract** | 1 | (*a*) Indicate the study’s design with a commonly used term in the title or the abstract | 02 | Abstract |
|  |  | (*b*) Provide in the abstract an informative and balanced summary of what was done and what was found | 02 | Methods and results |
| Introduction | | | |  |
| Background/rationale | 2 | Explain the scientific background and rationale for the investigation being reported | 03 | Chronic pain can affect physical performance by limiting the functional activities |
| Objectives | 3 | State specific objectives, including any prespecified hypotheses | 03 | Objective: it is to compare physical performance in women with and without endometriosis. Hypotheses: Understanding the impact of endometriosis on physical performance can help identify at-risk groups for disability and guide prevention and rehabilitation strategies. |
| Methods | | | |  |
| Study design | 4 | Present key elements of study design early in the paper | 04 | A cross-sectional study was conducted |
| Setting | 5 | Describe the setting, locations, and relevant dates, including periods of recruitment, exposure, follow-up, and data collection | 04 | Setting: at the outpatient clinic of the Januário Cicco School Maternity Hospital (MEJC). Locations: Federal University of Rio Grande do Norte (UFRN), Brazil. Relevant dates, including periods of recruitment and data collection: The study sample consisted of users of the outpatient clinic of MEJC/UFRN attended in the sector from September 2022 to March 2023.  Exposure and follow-up: not applicable |
| Participants | 6 | (*a*) *Cohort study*—Give the eligibility criteria, and the sources and methods of selection of participants. Describe methods of follow-up  *Case-control study*—Give the eligibility criteria, and the sources and methods of case ascertainment and control selection. Give the rationale for the choice of cases and controls  *Cross-sectional study*—Give the eligibility criteria, and the sources and methods of selection of participants | 04 | Both groups included women between 20 and 40 years old, with cognitive ability to understand and answer the questionnaires, as observed by the evaluator. Participants in the endometriosis group should have a diagnosis of endometriosis identified by clinical findings and confirmed by the presence of deep infiltration by magnetic resonance imaging (MRI). Specifically for the comparator group, women should have regular menstrual cycles and be in the late luteal phase for physical performance tests. |
|  |  | (*b*) *Cohort study*—For matched studies, give matching criteria and number of exposed and unexposed  *Case-control study*—For matched studies, give matching criteria and the number of controls per case | - | Not applicable |
| Variables | 7 | Clearly define all outcomes, exposures, predictors, potential confounders, and effect modifiers. Give diagnostic criteria, if applicable | 04/05/06 | Dependent variables: physical performance tests (Page 04), Covariates (05) and Other variables (06). |
| Data sources/ measurement | 8* | For each variable of interest, give sources of data and details of methods of assessment (measurement). Describe comparability of assessment methods if there is more than one group | 04/05/06 | Dependent variables: physical performance tests (Page 04), Covariates (05) and Other variables (06). |
| Bias | 9 | Describe any efforts to address potential sources of bias | 04 | Specifically for the comparator group, women should have regular menstrual cycles and be in the late luteal phase for physical performance tests. |
| Study size | 10 | Explain how the study size was arrived at | 04 | The study sample consisted of users of the outpatient clinic of MEJC/UFRN attended in the sector from September 2022 to March 2023. |

Continued on next page

| Quantitative variables | 11 | Explain how quantitative variables were handled in the analyses. If applicable, describe which groupings were chosen and why | - | Not applicable |
| --- | --- | --- | --- | --- |
| Statistical methods | 12 | (*a*) Describe all statistical methods, including those used to control for confounding | 06/07 | Statistical analysis topic |
|  |  | (*b*) Describe any methods used to examine subgroups and interactions | 06/07 | Statistical analysis topic |
|  |  | (*c*) Explain how missing data were addressed | - | Not applicable |
|  |  | (*d*) *Cohort study*—If applicable, explain how loss to follow-up was addressed  *Case-control study*—If applicable, explain how matching of cases and controls was addressed  *Cross-sectional study*—If applicable, describe analytical methods taking account of sampling strategy | - | Not applicable |
|  |  | (*e*) Describe any sensitivity analyses | - | Not applicable |
| Results | | | | |
| Participants | 13* | (a) Report numbers of individuals at each stage of study—eg numbers potentially eligible, examined for eligibility, confirmed eligible, included in the study, completing follow-up, and analysed | 07 | See table |
|  |  | (b) Give reasons for non-participation at each stage | - | Not applicable |
|  |  | (c) Consider use of a flow diagram | - | Not applicable |
| Descriptive data | 14* | (a) Give characteristics of study participants (eg demographic, clinical, social) and information on exposures and potential confounders | 07 | Table 1 |
|  |  | (b) Indicate number of participants with missing data for each variable of interest | - | There is not. |
|  |  | (c) *Cohort study*—Summarise follow-up time (eg, average and total amount) | - | Not applicable |
| Outcome data | 15* | *Cohort study*—Report numbers of outcome events or summary measures over time | - | Not applicable |
|  |  | *Case-control study—*Report numbers in each exposure category, or summary measures of exposure | - | Not applicable |
|  |  | *Cross-sectional study—*Report numbers of outcome events or summary measures | 07 | All participants in the EG were receiving pharmacological treatment for endometriosis (dienogest), and a median score of 6.40 (4.70 - 7.75) was observed for pain evaluation. |
| Main results | 16 | (*a*) Give unadjusted estimates and, if applicable, confounder-adjusted estimates and their precision (eg, 95% confidence interval). Make clear which confounders were adjusted for and why they were included | 07 | Table 3 |
|  |  | (*b*) Report category boundaries when continuous variables were categorized | 07 | Table 1 |
|  |  | (*c*) If relevant, consider translating estimates of relative risk into absolute risk for a meaningful time period | - | Not applicable |

Continued on next page

| Other analyses | 17 | Report other analyses done—eg analyses of subgroups and interactions, and sensitivity analyses | - | Not applicable |
| --- | --- | --- | --- | --- |
| Discussion | | | | |
| Key results | 18 | Summarise key results with reference to study objectives | 08/09 | Our results show that endometriosis is associated with worse physical performance in all tests considered, even after controlling by potential confounders. |
| Limitations | 19 | Discuss limitations of the study, taking into account sources of potential bias or imprecision. Discuss both direction and magnitude of any potential bias | 09 | Strength and Limitation Topic |
| Interpretation | 20 | Give a cautious overall interpretation of results considering objectives, limitations, multiplicity of analyses, results from similar studies, and other relevant evidence | 09/10 | Strength and Limitation Topic and Conclusion topic |
| Generalisability | 21 | Discuss the generalisability (external validity) of the study results | 08/09/10 | Discussion, Strength and Limitation and Conclusion topic |
| Other information | |  | | |
| Funding | 22 | Give the source of funding and the role of the funders for the present study and, if applicable, for the original study on which the present article is based | - | Not applicable |

*Give information separately for cases and controls in case-control studies and, if applicable, for exposed and unexposed groups in cohort and cross-sectional studies.

**Note:** An Explanation and Elaboration article discusses each checklist item and gives methodological background and published examples of transparent reporting. The STROBE checklist is best used in conjunction with this article (freely available on the Web sites of PLoS Medicine at http://www.plosmedicine.org/, Annals of Internal Medicine at http://www.annals.org/, and Epidemiology at http://www.epidem.com/). Information on the STROBE Initiative is available at www.strobe-statement.org.
